# p24^G1^ Encoded by Grapevine Leafroll-Associated Virus 1 Suppresses RNA Silencing and Elicits Hypersensitive Response-Like Necrosis in *Nicotiana* Species

**DOI:** 10.3390/v12101111

**Published:** 2020-09-30

**Authors:** Chen-Wei Zhang, Qing Liu, Qi Zeng, Wen-Ting Huang, Qi Wang, Yu-Qin Cheng

**Affiliations:** 1 Department of Pomology, China Agricultural University, Beijing 100193, China; cwzhang4192@foxmail.com (C.-W.Z.); 15838340768@163.com (Q.L.); qizeng360781@163.com (Q.Z.); cauwthuang@foxmail.com (W.-T.H.); 2 Key Laboratory of Viticulture and Enology, Ministry of Agriculture, Beijing 100083, China; 3 Department of Plant Pathology, China Agricultural University, Beijing 100193, China; wangqi@cau.edu.cn

**Keywords:** grapevine leafroll-associated virus 1, p24^G1^, RNA-silencing suppressor, pathogenicity, nuclear localization, self-interaction, siRNA binding

## Abstract

Grapevine leafroll-associated virus 1 (GLRaV-1) is a major pathogen associated with grapevine leafroll disease. However, the molecular mechanisms underlying GLRaV-1 interactions with plant cells are unclear. Using *Agrobacterium* infiltration-mediated RNA-silencing assays, we demonstrated that GLRaV-1 p24 protein (p24^G1^) acts as an RNA-silencing suppressor (RSS), inhibiting local and systemic RNA silencing. Electrophoretic mobility shift assays showed that p24^G1^ binds double-stranded 21-nucleotide small interfering RNA (siRNA), and that siRNA binding is required but not sufficient for its RSS activity. p24^G1^ localizes in the nucleus and can self-interact through its amino acid 10 to 210 region. Dimerization is needed for p24^G1^ interaction with importin α1 before moving to the nucleus, but is not required for its siRNA binding and RSS activity. Expression of p24^G1^ from a binary pGD vector or potato virus X-based vector elicited a strong hypersensitive response in *Nicotiana* species, indicating that p24^G1^ may be a factor in pathogenesis. Furthermore, p24^G1^ function in pathogenesis required its RSS activity, dimerization and nuclear localization. In addition, the region of amino acids 122–139 played a crucial role in the nuclear import, siRNA binding, silencing suppression and pathogenic activity of p24^G1^. These results contribute to our understanding of the molecular mechanisms underlying GLRaV-1 infection.

## 1. Introduction

RNA silencing is considered to be the most effective antiviral mechanism in plants. Antiviral RNA silencing is triggered by double-stranded (ds) RNAs that are recognized and processed by the RNaseIII-type DICER enzymes into 21–24 nucleotide (nt) small interfering RNA (siRNA) duplexes. The siRNAs are protected from degradation by HUA enhancer 1-dependent methylation [1] and then recruited by argonaute (AGO) proteins to form an RNA-induced silencing complex (RISC), thereby initiating the sequence-specific degradation of target RNAs [2]. In plants, the RNA-silencing signal can spread systemically to trigger systemic antiviral responses. To counter these responses, most plant viruses encode at least one RNA-silencing suppressor (RSS). Viral RSSs act via two general mechanisms: sequestering siRNAs to prevent their entry into the RISC, or inhibiting the function of proteins involved in RNA silencing [3]. For instance, the 2b protein encoded by cucumber mosaic virus (CMV) inhibits RNA silencing by binding ds siRNA [4] and interfering with RISC activity through interaction with AGO proteins [5,6]. The RSS activity of p19 encoded by tomato bushy stunt virus (TBSV) depends on the binding of ds siRNAs to prevent their subsequent incorporation into RISC [7]. The HC-Pro of potyviruses blocks RNA silencing through direct binding and sequestration of ds siRNA [7], and hijacking of the methionine cycle through inhibition of two of its key enzymes to block siRNA methylation [8].

The relevance of viral RSSs’ properties—including dimerization and subcellular localization—and their suppression function has been studied previously. For example, many viral RSSs act as dimers to block RNA silencing [9,10,11,12,13], while the monomers of tomato golden mosaic virus AL2 and beet curly top virus L2 can also suppress RNA silencing [14]. Similarly, in some instances, the nuclear distribution of nuclear-localized viral RSSs is essential for their suppressive function [15,16], whereas the RSS activity of CMV 2b [17], garlic virus X (GVX) p15 [18] and chrysanthemum virus B (CVB) p12 [19] is not correlated with their nuclear distribution pattern.

Given the key role of RNA-silencing suppression for virus survival, many viral RSSs have been proposed to be pathogenicity or virulence factors in plants. Viral RSSs are reported to play important roles in disease synergism and therefore enhancement of potato virus X (PVX) virulence by coexpression with other viral RSSs is a common phenomenon [20,21]. Some viral RSSs can also act as an elicitor to induce *hypersensitive response* (HR) when transiently expressed by agroinfiltration (reviewed in García and Pallás [22]).

Grapevine leafroll disease (GLD) is one of the most economically important diseases of grapes worldwide [23]. So far, six distinct viral species, referred to as grapevine leafroll-associated virus 1 (GLRaV-1), GLRaV-2, GLRaV-3, GLRaV-4, GLRaV-7 and GLRaV-13 of the *Closteroviridae* family, have been reported to be associated with GLD. GLRaV-1, GLRaV-3, GLRaV-4 and GLRaV-13 have been assigned to the genus *Ampelovirus*, GLRaV-2 is a *Closterovirus*, and GLRaV-7 is a key member of the *Crinivirus*. GLRaV-1 is considered one of the most common GLRaV species associated with GLD; its genome is about 19 kb in size and encodes 10 putative open reading frames (ORFs) [24]. ORFs 1a and 1b encode two replication-associated proteins, and the other eight ORFs encode a small transmembrane protein of 7 kDa (p7), a heat-shock protein 70 homolog, a polypeptide of 55 kDa (p55), coat protein (CP), two divergent copies of the CP (CPd1 and CPd2) and two polypeptides of 21 kDa (p21) and 24 kDa (p24) with unknown function [24]. In the genus *Closterovirus*, citrus tristeza virus encodes three distinct RSSs: p20, p23 and CP [25]; beet yellow virus (BYV) and GLRaV-2 encode one RSS: p21 [26] and p24 [27], respectively. Within the genus *Ampelovirus*, GLRaV-3 encodes one RSS, p19.7 [28]; pineapple mealybug wilt associated virus-2, which is closely related to GLRaV-1 and GLRaV-3, encodes four RSSs—p20, CP, p22 and CPd [29]. However, no such functional protein activity has been described for GLRaV-1.

The key role of viral RSSs in successful infection and symptom development, and the great diversity in their sequences, structures and modes of suppression highlight the importance of identifying new RSSs and elucidating the mechanisms governing their suppressive actions. Here, we demonstrate that p24 encoded by GLRaV-1 (hereafter referred to as p24^G1^) is localized in the nucleus and acts as an RSS, possibly through binding ds siRNA. We also show that p24^G1^ is a factor in pathogenesis eliciting a strong HR-like response in *Nicotiana* species when it is expressed from a binary vector pGD or PVX-based vector. Moreover, we show that p24^G1^ forms a dimer in the nucleus, with aa 10–210 required for the self-interaction. Finally, we evaluate the potential contribution of p24^G1^ homodimerization to its importin α1-mediated nuclear localization, binding of ds siRNA, silencing suppression function and activity in pathogenesis.

## 2. Materials and Methods

### 2.1. Plant Materials and Growth Conditions, and Preparation of Plasmids

GFP-transgenic line 16c and wild type (wt) *N. benthamiana* plants were grown under controlled conditions at 23–25 °C with a 16-h light regime. Grapevine (*Vitis vinifera*) cv. Centennial Seedless plants naturally infected with GLRaV-1 were grown in the experimental fields.

Primers used in this study are listed in Table S1. The sequence of p24^G1^ was RT-PCR-amplified from RNA extracted from petioles of “Centennial Seedless” plants with primer pair F1/R1, and subcloned into pMD19-T to produce pMD–p24^G1^. pMD–p24^G1^ was then used to generate pMD–∆122–139 with primer pair F2/R2 using the QuickMutation^TM^ kit (Beyotime, China) according to the manufacturer’s protocol. The sequence of ∆122–139 was amplified from pMD–∆122–139. pMD–p24^G1^ was used to PCR-amplify sequences of p24^G1^, ∆1–9, ∆1–21, ∆1–39 and ∆194–210.

For plasmids used in the yeast two-hybrid system (YTHS), sequences of p24^G1^, ∆1–9, ∆1–21, ∆1–39, ∆122–139 and ∆194–210 were PCR-amplified with primer pairs F3/R3, F4/R3, F5/R3, F6/R3, F3/R3 and F3/R4, respectively. PCR products were digested with *Eco*RI/*Bam*HI and subcloned into the vectors pGBKT7 and pGADT7 (Clontech Laboratories) to produce pGBK–p24^G1^, pGAD–p24^G1^, pGBK–∆1-9, pGAD–∆1–9, pGBK–∆1–21, pGAD–∆1–21, pGBK–∆1–39, pGAD–∆1–39, pGBK–∆122–139, pGAD–∆122–139, pGBK–∆194–210 and pGAD–∆194–210.

For the bimolecular fluorescence complementation (BiFC) assay, sequences of p24^G1^, ∆1–9, ∆1–21, ∆1–39, ∆122–139 and ∆194–210 were PCR-amplified with primer pairs F7/R5, F8/R5, F9/R5, F10/R5, F7/R5 and F7/R6, respectively. PCR products were digested with *Bam*HI/*Xho*I and subcloned into pSPYNE–35S and pSPYCE–35S [30] for expression of p24^G1^–YFP^N^, p24^G1^–YFP^C^, ∆1–9–YFP^N^, ∆1–9–YFP^C^, ∆1–21–YFP^N^, ∆1–21–YFP^C^, ∆1–39–YFP^N^, ∆1–39–YFP^C^, ∆122–139–YFP^N^, ∆122–139–YFP^C^, ∆194–210–YFP^N^ and ∆194–210–YFP^C^.

For the RNA-silencing suppression assay, sequences of p24^G1^, ∆1–9, ∆1–21, ∆1–39, ∆122–139 and ∆194–210 were PCR-amplified with primer pairs F11/R7, F12/R7, F13/R7, F14/R7, F11/R7 and F11/R8, respectively. PCR products were digested with *Xho*I/*Bam*HI and subcloned into pGD [31] to produce pGD–p24^G1^, pGD–∆1–9, pGD–∆1–21, pGD–∆1–39, pGD–∆122–139 and pGD–∆194–210.

For the subcellular localization assay, sequences of p24^G1^, ∆1–9, ∆1–21, ∆1–39, ∆122–139 and ∆194–210 were PCR-amplified with primer pairs F15/R9, F16/R9, F17/R9, F18/R9, F15/R9, F15/R10 and F19/R11, respectively. PCR products were digested with *Sac*I/*Bam*HI and subcloned into pCam35s–GFP for expression of p24^G1^–GFP, ∆1–9–GFP, ∆1–21–GFP, ∆1–39–GFP, ∆122–139–GFP and ∆194–210–GFP. PCR products of p24^G1^ were also digested with *Xho*I/*Bam*HI and subcloned into pGDG [31] for expression of GFP–p24^G1^.

For construction of recombinant PVX vectors, sequences of p24^G1^, ∆1–9, ∆1–21, ∆1–39, ∆122–139 and ∆194–210 were PCR-amplified with primer pairs F20/R12, F21/R12, F22/R12, F23/R12, F20/R12 and F20/R13, respectively. PCR products were digested with *Cla*I/*Sma*I and subcloned into PVX vector [32] to produce PVX–p24^G1^, PVX–∆1–9, PVX–∆1–21, PVX–∆1–39, PVX–∆122–139, and PVX–∆194–210.

Sequences of *N. benthamiana* fibrillarin 2 (No. FM244835.1) and importin α1 (No. EF137253.1) were RT-PCR-amplified from RNA extracted from *N. benthamiana* leaves with primer pairs F24/R14 and F25/R15. The former PCR products were digested with *Xho*I/*Bam*HI and subcloned into pGDR [31] to produce pRFP–fibrillarin. The latter were digested with *Bam*HI/*Xho*I and subcloned into pSPYNE–35S and pSPYCE–35S for expression of IMPα1–YFP^N^ and IMPα1–YFP^C^, respectively.

### 2.2. Agroinfiltration and Fluorescence Imaging

The young fully expanded leaves of *N. tabacum*, and wt and line 16c *N. benthamiana* plants were used for agroinfiltration. *Agrobacterium tumefaciens* GV3101 was transformed with each plasmid and agroinfiltration was performed according to previously described methods [27]. For RSS activity and pathogenicity assays, agroinfiltrated plants were monitored every day. The leaf samples from the same treatment within each experiment were mixed for extraction of total RNA and protein.

For BiFC and subcellular localization assays, 4’,6-diamidino-2-phenylindole (*DAPI*) and fluorescence signals were visualized at 3 dpi using an Olympus FluoView 1000 confocal microscope equipped with Olympus FluoView FV10-ASW 3.1 Viewer Software, or an Olympus FluoView 3000 confocal microscope equipped with FV31S-SW Viewer Software.

For the RSS activity assay, plants were illuminated with a 100 W handheld longwave ultraviolet (UV) lamp (UV Products, Upland, CA, USA; Black Ray model B 100AP/R), and GFP images were taken with a digital camera. Each experiment was repeated three times.

### 2.3. Northern Blot

Sequences of digoxigenin (DIG)-labeled cDNA probes used for detection of the GFP-derived siRNAs and U6 small nuclear RNA have been previously described [33], and probes were generated by Sangon Biotech company (China).

Northern blotting and generation of DIG-labeled cDNA probes for detection of PVX RNA and GFP mRNA were performed using the DIG High Prime DNA Labeling and Detection Starter Kit II (Roche, Basel, Germany) according to the manufacturer’s protocol. Sequences of probes for detection of PVX RNA or GFP mRNA corresponded to the PVX CP sequence or nt 62–673 of the GFP sequence, respectively.

RNA samples of 15 or 30 µg were used to detect high-molecular weight RNA or siRNA. Total RNA was separated on 1% agarose-formaldehyde gels, transferred to Hybond-N+ membranes and hybridized with DIG-labeled probes.

Each assay was repeated in three independent experiments.

### 2.4. Protein Interaction Analysis in Yeast

The small-scale lithium acetate transformation method was performed according to the manufacturer’s protocol (Clontech Laboratories). Transformed yeast cells were plated on synthetic medium without leucine (Leu), tryptophan (Trp), histidine (His) and adenine (Ade) (SD/-Leu-Trp-Ade-His). Each experiment was repeated three times.

### 2.5. Protein Expression and Purification

The sequence of ∆122–139 was amplified from pMD–∆122–139 with primer pair F26/R16. Sequences of p24^G1^, ∆1–9, ∆1–21, ∆1–39 and ∆194–210 were amplified from pMD–p24^G1^ with primer pairs F26/R16, F27/R16, F28/R16, F29/R16 and F26/R17, respectively. The GFP sequence was amplified from pGDG [31] with primer pair F30/R18. PCR products were digested with *Bam*HI/*Xho*I and subcloned into vector pET28a to produce His-fusion constructs pET–p24^G1^, pET–∆1–9, pET–∆1–21, pET–∆1–39, pET–∆122–139, pET–∆194–210 and pET–GFP. These constructs were separately transformed into *E. coli* (BL21) for protein expression. His-tag fusion proteins were purified using His-bind-Trap (Novagen, Madison, Malaysia), according to the manufacturer’s instructions.

### 2.6. Plant Protein Extraction and Western Blot Analysis

Extraction of protein from plant tissues and Western blot analysis were performed as detailed previously [27]. Antibody/antisera and dilutions used in the Western blot analysis were as follows: anti-GFP antibody (1:5000, ComWin, Beijing, China), and anti-p24^G1^ (1:2000, noncommercial), anti-CP of PVX (1:2000, noncommercial) and anti-NbPR10 (1:1000, noncommercial) antisera. Finally, PVX CP was detected with the substrate 5-bromo-4-chloro-3-indolyl phosphate/nitroblue tetrazolium (Sigma, USA), and GFP, p24^G1^ and NbPR10 were detected using an enhanced chemiluminescence system (eECL Western Blot Kit) (ComWin, Beijing, China). An equal amount of protein content for each lane was visualized by Coomassie brilliant blue (CBB) staining.

### 2.7. Electrophoretic Mobility Shift Assay (EMSA)

EMSA experiments were performed using the LightShift^®^ Chemiluminescent EMSA Kit (Thermo Scientific, Carlsbad, CA, USA) according to the manufacturer’s instructions. Biotin-labeled single-stranded RNA oligos synthesized by Sangon Biotech (Shanghai, China) were annealed to form duplex sRNA probes. Annealing was performed as described [34]. Sequences of probes sRNA-1/sRNA-2 (21 nt) [34] are indicated in Table S1.

A constant amount (10 ng) of probes was incubated with 0.05 or 0.5 μg of His-tagged fusion protein. Incubation with GFP–His was used as a negative control. Biotin-labeled duplex sRNA was detected by chemiluminescence. Each experiment was repeated three times.

### 2.8. Cell Death Analysis and H_2_O_2_ Detection

For Trypan blue or 3,3′-diaminobenzidine (DAB) staining, the leaves were placed in the Trypan blue (10 mL lactic acid, 10 mL glycerol, 10 mL phenol, 10 mL double-distilled H_2_O and 15 mg Trypan blue) or DAB (1 mg/mL DAB, pH 5.7) staining solution for 5 h after slight vacuum infiltration, rinsed with double-distilled H_2_O and then boiled in a 95% (*v*/*v*) ethanol solution for 10 min. Each assay was conducted independently three times.

### 2.9. RT-PCR, qRT-PCR and Statistical Analysis

Total RNA was isolated from *N. benthamiana* leaves or grapevine petioles using the RNeasy Plant Mini Kit (Qiagen, Dusseldorf, North Rhine-Westphalia, Germany). Synthesis of cDNA (at 42 °C) was primed with a mix of random primers using 500 ng of total RNA.

PCR amplification was conducted as follows: cDNA denaturation at 95 °C for 5 min; 35 cycles at 94 °C for 30 s, 52–55 °C (depending on the specific primer pair used) for 30 s and 72 °C for 45–60 s, and a final extension step at 72 °C for 10 min.

qRT-PCR was performed with the SYBR^®^ PrimeScript™ RT-PCR Kit (TAKARA, Beijing, China) according to the manufacturer’s instructions. The primer pairs for PVX *CP*, *NbPR1* (No. JN247448.1), *NbPR4* (No. XM_019370073.1) and *NbPR10* (No. KF841443.1) were F31/R19, F32/R20, F33/R21 and F34/R22, respectively. *N. benthamiana GAPDH* (No. AB937979.1) was analyzed as an internal control using primer pair F35/R23. qPCR analysis was conducted in an ABI 7500 thermocycler (Applied Biosystems, Carlsbad, CA, USA). Quantification was conducted according to the method described by Pfaffl [35].

Data are presented as means and standard deviations. Significant differences between the treatments and the controls were determined using the statistical software package SPSS, which was also used for *statistical* analyses (*SPSS* Inc., Chicago, IL, USA, 2001).

## 3. Results

### 3.1. p24^G1^ Suppresses Local and Systemic RNA Silencing

**Figure 1:**
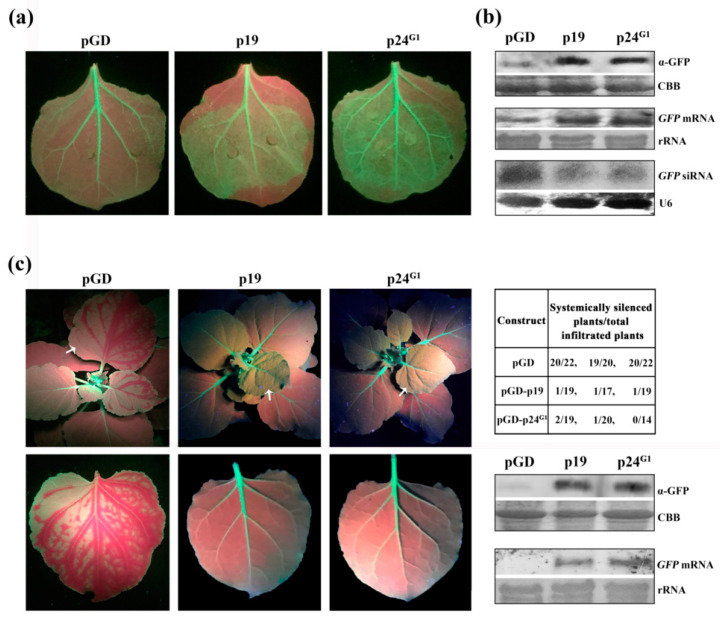
Suppression by p24 of grapevine leafroll-associated virus 1 (p24^G1^) of local and systemic RNA silencing. (**a**) GFP image of agroinfiltrated patches of *N. benthamiana* line 16c under UV at 3 dpi. Each indicated GFP image is representative of more than 20 individual leaf samples from three separate experiments. (**b**) Quantitative assessment of GFP protein and mRNA, and GFP-specific siRNA accumulation. Total protein and RNA were extracted from the agroinfiltrated patches at 3 dpi. (**c**) Effect of p24^G1^ on systemic silencing. Photographs were taken under UV light at 18 dpi, and leaves indicated by arrows are shown in the panels below. The number of plants that showed systemic silencing out of the total number of plants tested, 18 dpi, is indicated in the table. The lower right panel shows GFP protein and mRNA accumulation, 18 dpi, in the distal leaves of infiltrated plants. Coinfiltration of pGD–GFP and the empty vector pGD or pGD–p19 was used as negative or positive controls (**a**,**c**). CBB staining (**b**,**c**), Ethidium-bromide-stained rRNA (**b**,**c**) and detection of U6 small nuclear RNA (**b**) are shown as loading controls.

Products of the ORFs in the 3’ region of viruses of the *Closteroviridae* family are suggested to be involved in the suppression of RNA silencing [36]. Based on a comparison of the genome organization of GLRaV-1, GLRaV-2 and GLRaV-3, p24^G1^ (GenBank Accession No. MN660142), encoded by the tenth ORF in the 3’-terminal region of the GLRaV-1 genome, was selected as a candidate RSS and cloned into the expression cassette of the binary vector pGD to explore its antisilencing activity. Leaves of GFP-expressing transgenic *N. benthamiana* line 16c [37] were infiltrated with cultures harboring pGD–GFP and pGD–p24^G1^. Coinfiltration of pGD–GFP with an empty vector (pGD), or with pGD–p19 [27] expressing p19 of TBSV, a well-characterized suppressor, was used as negative and positive controls, respectively. Leaf patches expressing p24^G1^ showed strong GFP fluorescence three days post-infiltration (dpi), similar to that with expression of p19 (Figure 1a). In contrast, GFP fluorescence was very weak in tissues coinfiltrated with pGD–GFP and pGD (Figure 1a). Moreover, Western blot and Northern blot analyses revealed that the strong GFP fluorescence in the sectors expressing p24^G1^ was directly correlated with higher levels of GFP protein and mRNA, and lower accumulation of GFP-derived siRNA, as compared to the negative controls (Figure 1b). These quantitative analyses confirmed the visual observations, indicating that p24^G1^ can suppress local RNA silencing.

RNA silencing originating at one site can *spread* over long distances to induce systemic RNA silencing. To evaluate whether GLRaV-1 p24 can interfere with systemic silencing, leaves of line 16c were agroinfiltrated with pGD–GFP/pGD–p24^G1^, and GFP silencing in the upper noninfiltrated leaves was monitored. The results of three independent experiments are summarized in the table in Figure 1c. In 55% of the negative control plants (coinfiltrated with pGD–GFP and pGD), the upper newly emerging leaves started to lose GFP fluorescence between major veins as early as 8–10 dpi, and at 18 dpi, about 92% of the negative control plants displayed vein-proximal silencing of GFP in newly emerging leaves (Figure 1c, left panel). In contrast, about 94% or 95% of the upper noninfiltrated leaves in plants coinfiltrated with pGD–GFP and pGD–p24^G1^ or pGD–p19 retained GFP fluorescence for 18 dpi (Figure 1c, left panel). Consistent with this, much higher levels of GFP protein and mRNA were observed in these leaves compared to negative control plants (Figure 1c, lower right panels), supporting the visual observation that silencing signals for the GFP transgene did not spread to the upper noninfiltrated leaves. 

Together, our results demonstrated that p24^G1^ suppresses local and systemic RNA silencing.

### 3.2. p24^G1^ Is a Factor in Pathogenesis Eliciting HR-Like Necrosis in Nicotiana Species

**Figure 2:**
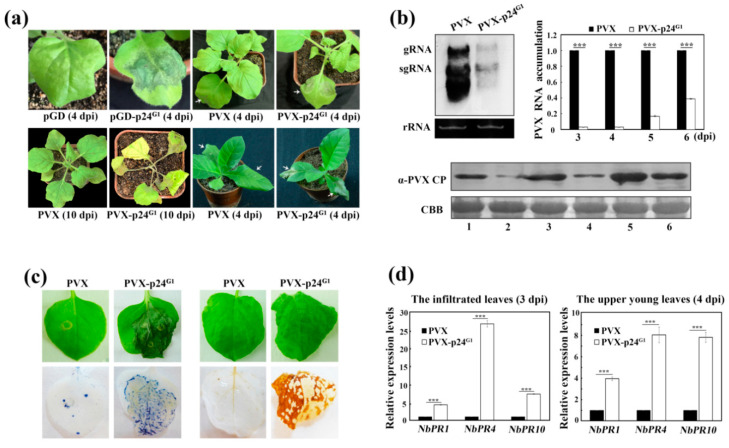
p24 of grapevine leafroll-associated virus 1 (p24^G1^) elicits necrosis in *Nicotiana* species when expressed from pGD or PVX vector. (**a**) Phenotypes of *N. benthamiana* and *N. tabacum* induced by p24^G1^ expressed from pGD- or PVX-based vector. Arrows indicate infiltrated leaves. (**b**) Quantitative assessment of PVX accumulation. The upper young leaves of PVX–p24^G1^- or PVX-infected *N. benthamiana* plants at 3, 4, 5 and 6 dpi were used for RNA and protein extraction. The upper left panel shows PVX RNA accumulation at 5 dpi detected by Northern blot. The detected bands corresponding to PVX genomic and subgenomic RNA are indicated as gRNA and sgRNA, respectively. The upper right panel shows PVX RNA accumulation at 3, 4, 5 and 6 dpi detected by qRT-PCR. The lower panel shows PVX CP accumulation at 4 (lanes 1, 2), 5 (lanes 3, 4) and 6 (lanes 5, 6) dpi detected by Western blot using PVX CP-specific antiserum. Odd and even lane numbers indicate samples from PVX- and PVX–p24^G1^-infected plants, respectively. Ethidium-bromide-stained rRNA and CBB staining are shown as loading controls. (**c**) Trypan blue and DAB staining. Cell death was estimated by Trypan blue staining of infiltrated leaves at 4 dpi. H_2_O_2_ accumulation was assessed by DAB staining of upper noninfiltrated leaves at 5 dpi. Representative leaves before and after staining are shown. (**d**) Effect of p24^G1^ expressed from PVX vector on the transcript levels of *NbPR1*, *NbPR4* and *NbPR10*. Total RNA was extracted from the infiltrated (3 dpi) and upper young (4 dpi) leaves. qRT-PCR results (b,d) are shown as mean  ±  standard deviation of three independent experiments. Standard deviation is denoted by error bars. *** *p* < 0.001.

During the *Agrobacterium* coinfiltration assay, we noticed that leaf patches of line 16c that coinfiltrated with pGD–p24^G1^ and pGD–GFP displayed local necrosis at 4 dpi, whereas coinfiltration with pGD and pGD–GFP did not produce a necrotic response (Figure S1), suggesting that p24^G1^ plays a role in *pathogenicity*. To further evaluate this, leaves of wt *N. benthamiana* plants were infiltrated with pGD–p24^G1^ alone. As expected, the agroinfiltrated tissues developed local necrosis at 4 dpi (Figure 2a), resembling the phenotype observed in the coinfiltration assay (Figure S1).

p24^G1^ was then expressed from a PVX-based vector. As shown in Figure 2a, expression of p24^G1^ substantially enhanced the virulence and pathogenicity of the recombinant virus PVX–p24^G1^: *N. benthamiana* plants infiltrated with PVX–p24^G1^ showed local necrosis in infiltrated tissues at 4 dpi, and at 7 dpi, the plants showed typical apical necrosis that ultimately led to the death of the entire plant, at 10 dpi. In contrast, PVX-infected plants only showed mild mosaic symptoms. Western blot and RT-PCR results confirmed the expression of p24^G1^ in the upper noninfiltrated *N. benthamiana* leaves before necrosis, at 4 dpi (Figure S2), suggesting that p24^G1^ was accurately maintained in the viral progeny. In addition, PVX–p24^G1^ elicited local necrosis, covering the infiltrated patches of *N. tabacum* plants at 4 dpi (Figure 2a, rightmost lower panel).

To analyze whether the enhanced pathogenicity of PVX–p24^G1^ is due to an increase in the PVX titer, PVX accumulation in the upper young leaves of PVX–p24^G1^-infected *N. benthamiana* plants was determined at 3, 4, 5 and 6 dpi. Both Northern blot (Figure 2b, upper left panel) and qRT-PCR (Figure 2b, upper right panel) results showed that PVX RNA accumulation in PVX–p24^G1^-infected plants is much lower than in their PVX-infected counterparts. Western blotting results also showed low accumulation of PVX CP in PVX–p24^G1^-infected plants (Figure 2b, lower panel). These results indicated that exacerbation of the symptoms caused by PVX–p24^G1^ was not linked to an increase in PVX accumulation.

To investigate whether the necrosis triggered by p24^G1^ shares HR characteristics, cell death, the accumulation of H_2_O_2_ and induction of defense-related genes were analyzed. At 4 dpi, the PVX–p24^G1^-infiltrated *N. benthamiana* leaves were deeply stained by treatment with Trypan blue, while the PVX-infected leaves were only lightly stained (Figure 2c), suggesting the occurrence of cell death in response to p24^G1^. We also observed that, at 5 dpi, the upper noninfiltrated leaves of PVX–p24^G1^-infected plants produced a deep brown color after DAB staining, indicating strong accumulation of H_2_O_2_ (Figure 2c).

Pathogenesis-related (PR) protein genes *PR1*, *PR4* and *PR10* have been reported to be involved in plant cell death and defense responses [38,39]. The qRT-PCR results revealed that transcript levels of *NbPR1*, *NbPR4* and *NbPR10* in the infiltrated leaves (3 dpi) and upper young leaves (4 dpi) of PVX–p24^G1^-infected plants, before necrosis, were all much higher than in PVX-infected plants (Figure 2d). 

Taken together, our results indicated that p24^G1^ is a factor in pathogenesis eliciting HR-like necrosis in *Nicotiana* species.

### 3.3. p24^G1^ Self-Interacts in the Nucleus Through Its 10–210 aa Region

**Figure 3:**
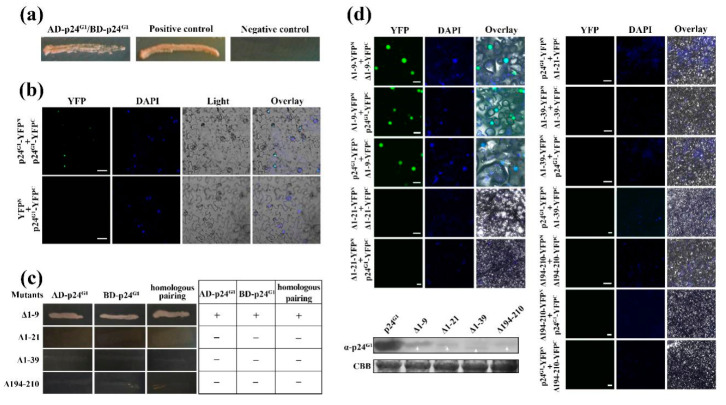
Functional region responsible for self-interaction of p24 of grapevine leafroll-associated virus 1 (p24^G1^). (**a**,**b**) p24^G1^ self-interacts in yeast (**a**) and plant (**b**) cells. Yeast strain AH109 cells coexpressing AD–p24^G1^ and BD–p24^G1^ were plated on SD/-Leu-Trp-His-Ade plates. Pairs of pGBKT7/pGAD–p24^G1^ and pGBKT7–53/pGADT7–RecT (provide by the kit) served as negative and positive controls, respectively. p24^G1^–YFP^N^/p24^G1^–YFP^C^ were coexpressed in *N. benthamian*a leaves. Coexpression of YFP^N^/p24^G1^–YFP^C^ served as the negative control. Bars = 50 μm. (**c**,**d**) Analyses of self-interaction of p24^G1^ mutants as well as the interaction between mutants and wt p24^G1^ in yeast (**c**) and plant (**d**) cells. The indicated mutants were fused to GAL4 AD and BD, respectively (**c**). A summary of interaction results in yeast is indicated in the table. +, interaction; −, no interaction. Bars = 100 μm (scale bars in the images marked with “∆1–9” are 20 μm). The expression of wt p24^G1^ and mutants fused to the YFP^N^ in plant cells at 3 dpi was monitored by Western blot as indicated in the lower left panel. CBB staining served as a loading control. Nuclei of tobacco leaf epidermal cells were stained with DAPI (**b**,**d**).

To assess whether p24^G1^ can interact with itself, the p24^G1^ sequence was fused with the GAL4 activation domain (AD) in pGADT7 or the DNA-binding domain (BD) in pGBKT7 in YTHS. Yeast transformants carrying pGBK–p24^G1^ and pGAD–p24^G1^ were able to grow on SD/-Leu-Trp-Ade-His medium, whereas negative controls (yeast transformants carrying pGBKT7/pGAD–p24^G1^) could not (Figure 3a), indicating that p24^G1^ can form a dimer in yeast cells.

The BiFC assay was used to investigate whether p24^G1^ can self-interact in planta. The p24^G1^ sequence was fused to YFP^N^ (N terminus of YFP) in pSPYNE–35S and YFP^C^ (C terminus of YFP) in pSPYCE–35S vectors, respectively. As shown in Figure 3b, strong YFP fluorescence was observed in the nucleus of *N. benthamiana* cells coexpressing p24^G1^–YFP^N^ and p24^G1^–YFP^C^, which colocalized with *nuclear DAPI* staining. In contrast, no or negligible fluorescence was observed in the leaves coexpressing p24^G1^–YFP^C^/YFP^N^.

Secondary structure analysis (http://bioinf.cs.ucl.ac.uk/psipred/) predicted that p24^G1^ has nine α-helices located between aa 12 and 207, and six β-strands located between aa 64 and 201 (Figure S3a). Thus, four truncated derivatives (Figure S3b): ∆1–9 (deletion of aa 1–9), ∆1–21, ∆1–39 and ∆194–210, were created to map the functional region necessary for dimerization of p24^G1^. Self-interaction of truncated mutants, as well as interactions between truncated mutants and wt p24^G1^ were tested by YTHS. The results of three independent experiments are summarized in Figure 3c. In both homologous pairings and pairings with wt p24^G1^, only mutant ∆1–9 was able to self-interact and interact with wt p24^G1^ in yeast cells.

The BiFC assay also showed that only ∆1–9 can self-interact and interact with wt p24^G1^ in the nucleus of plant cells (Figure 3d, right and upper left panels). Western blotting results demonstrated the expression of all YFP^N^-tagged mutants in plant cells (Figure 3d, lower left panel), suggesting that the lack of self-interaction is not due to loss of expression. However, the expression of these mutants was obviously weaker than that of wt p24^G1^, indicating that mutations affect the protein’s stability.

Taken together, our results indicated that p24^G1^ self-interacts in the nucleus, and that aa 10–210, a region that includes all putative α-helices and β-strands, is required for dimer formation.

### 3.4. Dimerization of p24^G1^ Is a Prerequisite for Its Nuclear Targeting Mediated by Importin α1

**Figure 4:**
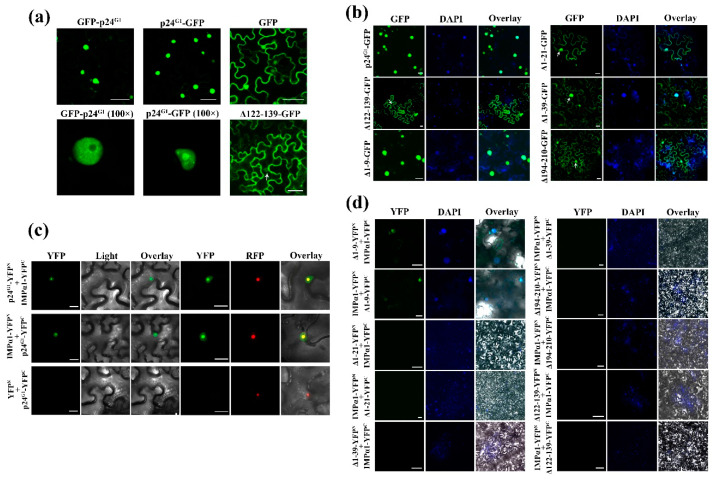
p24 of grapevine leafroll-associated virus 1 (p24^G1^) is localized in the nucleus and interacts with importin α1. (**a**) Confocal micrographs show the nuclear localization of fusion proteins p24^G1^–GFP and GFP–p24^G1^ in epidermal cells of *N. benthamiana* leaves. Bars = 50 μm. (**b**) Subcellular localization of p24^G1^ mutants. Bars = 20 μm. (**c**) p24^G1^ interacts with *N. benthamiana* importin α1 (NbIMPα1) in the nucleolus. RFP-tagged fibrillarin of *N. benthamiana* was used as a nucleolar protein control. Bars = 20 μm. (**d**) Analysis of the interaction between p24^G1^ mutants and NbIMPα1. Bars =100 μm (scale bars for the images marked with “∆1–9” are 20 μm). Arrows indicate the nucleus (**a**,**b**). Nuclei of tobacco leaf epidermal cells were stained with DAPI (**b**,**d**).

Our observation that p24^G1^ forms a dimer in the nucleus (Figure 3c) suggested its potential nuclear localization. To further clarify the subcellular localization of p24^G1^, p24^G1^–GFP and GFP–p24^G1^ (GFP fused to the C or N terminus of p24^G1^, respectively) were expressed in *N. benthamiana* leaves via agroinfiltration. Confocal laser scanning microscopy of the leaves at 3 dpi revealed that GFP fluorescence derived from both fusion proteins preferentially accumulates in the nucleus, with a very *weak cytoplasmic* distribution (Figure 4a). Within the former organelle, predominantly nucleolus localization was further observed for p24^G1^–GFP but not GFP–p24^G1^ using a 100× oil objective (Figure 4a, lower middle and left panels). The distribution pattern of GFP-tagged p24^G1^ differed from that of free GFP, which showed the typical cytoplasmic and nuclear distribution (Figure 4a).

*Bioinformatics analysis* (http://www.moseslab.csb.utoronto.ca/NLStradamus) predicted a possible nuclear localization signal (NLS) in the aa 122–139 region of p24^G1^ (Figure S4a, upper panel), which is rich in basic residues (122-RDRKKKGFSRTLLKRVTKA-139). Therefore, p24^G1^ mutant ∆122–139 (deletion of aa 122–139; Figure S4a, lower panel) was generated and inserted into pCam35S–GFP for expression of ∆122–139–GFP. ∆122–139–GFP accumulated mainly in the cytoplasm, with only a very weak GFP signal in the nucleus of *N. benthamiana* cells (Figure 4a, lower right panel); this was further confirmed by DAPI staining (Figure 4b). The same approach was also employed to analyze the subcellular localization of other p24^G1^ mutants: ∆1–9, ∆1–21, ∆1–39 and ∆194–210, which retained the predicted NLS. The subcellular localization of ∆1–9 with self-interaction ability was essentially identical to that of wt p24^G1^, whereas the other three mutants, which lacked dimerization ability, were almost evenly distributed in the cytoplasm and nucleus (Figure 4b). In addition, we observed that ∆122–139 lost homologous interactions in both yeast and plant cells, although the YFP^N^-tagged ∆122–139 was expressed at a level similar to that of YFP^N^–p24^G1^ (Figure S4b,c). Therefore, our results confirmed the presence of an NLS in the region of aa 122–139 and indicated that the nuclear localization of p24^G1^ requires its formation of a dimer.

The classical nuclear import pathway depends on importin α [40]. We investigated the possible interaction between p24^G1^ and *N*. *benthamiana* importin α1 (NbIMPα1; No. EF137253.1) by BiFC. Reconstitution of YFP fluorescence was observed in the nucleus of cells in *N. benthamiana* leaves coexpressing p24^G1^–YFP^N^/IMPα1–YFP^C^ or p24^G1^–YFP^C^/IMPα1–YFP^N^ (Figure 4c). More specifically, the interaction was observed mainly in the nucleolus, which colocalized with the red fluorescence protein (RFP)-tagged nucleolar *protein* fibrillarin of *N. benthamiana* (Figure 4c). In contrast, no fluorescence was observed in the negative controls (leaves coinfiltrated with YFP^N^/p24^G1^–YFP^C^). These results indicated that p24^G1^ nuclear targeting is mediated by importin α.

Since nuclear localization of p24^G1^ requires its self-interaction, we then assessed whether dimerization is necessary for p24^G1^ interaction with NbIMPα1. As shown in Figure 4d, YFP fluorescence was observed in the nucleus of *N. benthamiana* leaves coexpressing ∆1–9–YFP^N^/IMPα1–YFP^C^ or ∆1–9–YFP^C^/IMPα1–YFP^N^. In contrast, no interaction was detected between NbIMPα1 and p24^G1^ mutants lacking the homologous interaction, i.e., ∆1–21, ∆1–39, ∆194–210 and ∆122–139 (Figure 4d). Thus, our results suggested that dimerization is required for p24^G1^ interaction with importin α1 before moving to the nucleus.

### 3.5. p24^G1^ Is Able to Bind ds siRNA

**Figure 5:**
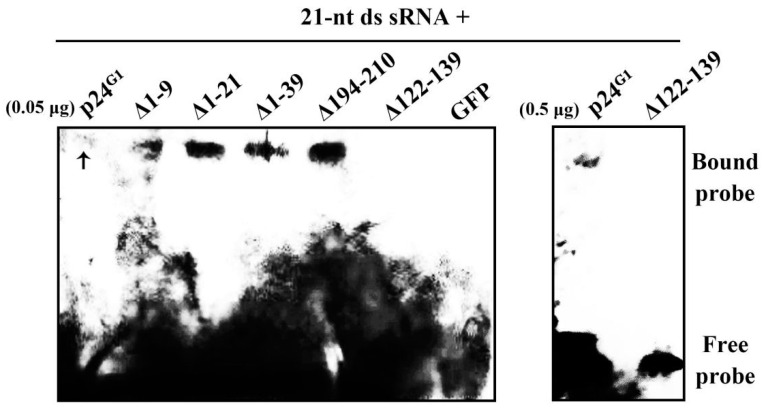
EMSA of the binding of p24 of grapevine leafroll-associated virus 1 (p24^G1^) and mutants to 21-nt sRNA duplexes. A constant amount (10 ng) of biotin-labeled 21-nt sRNAs was incubated with 0.05 or 0.5 μg of the indicated protein with his-tagged. GFP–His served as the negative control. Arrow indicates the p24^G1^–sRNA complex.

Viral RSSs generally adopt a ds siRNA-binding mechanism to block RNA silencing [7]. To assess whether p24^G1^ shares this strategy, EMSA was conducted. His-tagged p24^G1^ (p24^G1^–His) was incubated with 21-nt sRNA duplexes. The results showed that p24^G1^–His is able to bind sRNA duplexes, albeit very weakly (Figure 5). In contrast, the negative control GFP–His protein failed to form complexes with sRNA duplexes. Mutants ∆1–9, ∆1–21, ∆1–39 and ∆194–210 were all able to bind to the 21-nt sRNA duplexes (Figure 5), regardless of whether they could form dimers, suggesting that p24^G1^ can bind ds siRNA as a monomer. The yield of purified His-tagged mutants obtained from the *E.* *coli* expression system was too low to be visualized by protein staining after SDS-PAGE (Figure S5). However, they bound 21-nt sRNA duplexes more effectively than the wt p24^G1^: the binding of p24^G1^–His to probes was almost undetectable under the same experimental conditions (Figure 5, left panel), and the shifted banding was still weak when a higher amount of p24^G1^–His (0.5 μg) was used (right panel).

In addition, ∆122–139–His without the predicted NLS failed to bind sRNA duplexes (Figure 5), even though its purified yield obtained from the *E*. *coli* expression system was similar to that of wt p24^G1^ (Figure S5). These results indicated that the aa 122–139 region is involved in p24^G1^ binding to ds siRNA.

### 3.6. Monomeric p24^G1^ Can Suppress RNA Silencing, and siRNA Binding Is Insufficient for Its RSS Activity

**Figure 6:**
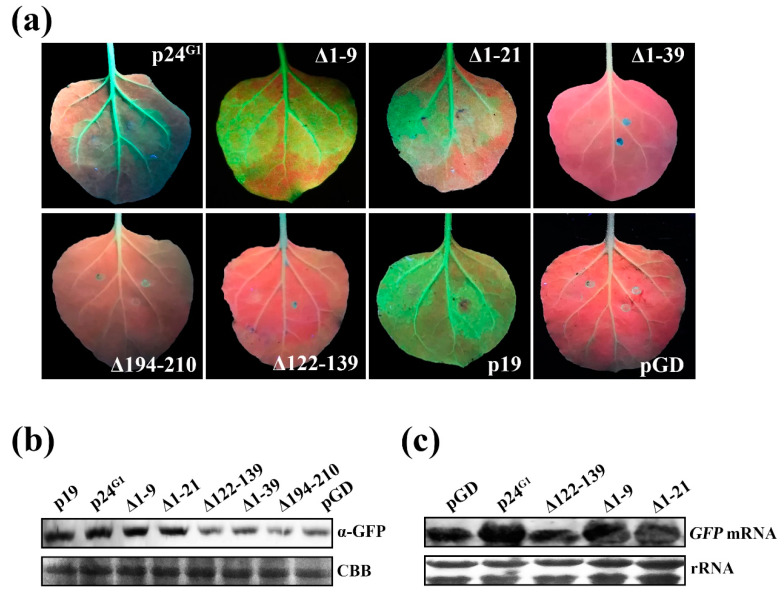
Analysis of silencing suppression activity of p24 of grapevine leafroll-associated virus 1 (p24^G1^) mutants. (**a**) GFP images of agroinfiltrated patches of *N. benthamiana* line 16c under UV at 3 dpi, infiltrated with a mixture of *Agrobacterium* cultures containing pGD–GFP and a plasmid expressing the indicated proteins. Each indicated GFP image is representative of more than 15 individual leaf samples from three separate experiments. Coinfiltration of pGD–GFP and the empty vector pGD or pGD–p19 was used as negative or positive control. (**b**) GFP protein accumulation detected by Western blot using anti-GFP antibody. (**c**) Northern blot analysis of GFP mRNA using DIG-labeled probes. Total protein (**b**) and RNA (**c**) were extracted from the agroinfiltrated patches at 3 dpi. CBB staining (**b**) and Ethidium-bromide-stained rRNA (**c**) served as loading controls.

To evaluate the relevance of the self-interaction for the suppression activity of p24^G1^, mutant ∆1–9 with dimerization ability, and the dimerization-defective mutants ∆1–21, ∆1–39, ∆194–210 and ∆122–139 were selected to assess their RSS activity. Leaf patches of line 16c expressing ∆1–9 or ∆1–21 displayed intense GFP fluorescence (Figure 6a), whereas only a weak GFP signal was observed on leaf patches expressing ∆122–139, ∆1–39 or ∆194–210, similar to the negative control (Figure 6a,b). Quantitative analysis of GFP protein by Western blot confirmed the visual observation (Figure 6b). Total RNA was extracted from leaf patches expressing GFP plus ∆1–9, ∆1–21 or ∆122–139 to further analyze the GFP mRNA level. Compared to the expression in ∆122–139-expressing leaf patches or negative controls, expression of ∆1–9 and ∆1–21 resulted in a high level of GFP mRNA (Figure 6c). Thus, our results demonstrated that ∆1–9 and ∆1–21 retain RSS activity, indicating that the aa 22–210 region is responsible for RSS activity and that self-interaction is not required for p24^G1^ suppression of RNA silencing. Since ∆1–39 and ∆194–210 can bind 21-nt sRNA duplexes (Figure 5), our results also indicated that ds siRNA binding is required but not sufficient for suppression by p24^G1^.

### 3.7. Pathogenic Activity of p24^G1^ Requires Both Its RSS Activity and Dimerization

**Figure 7:**
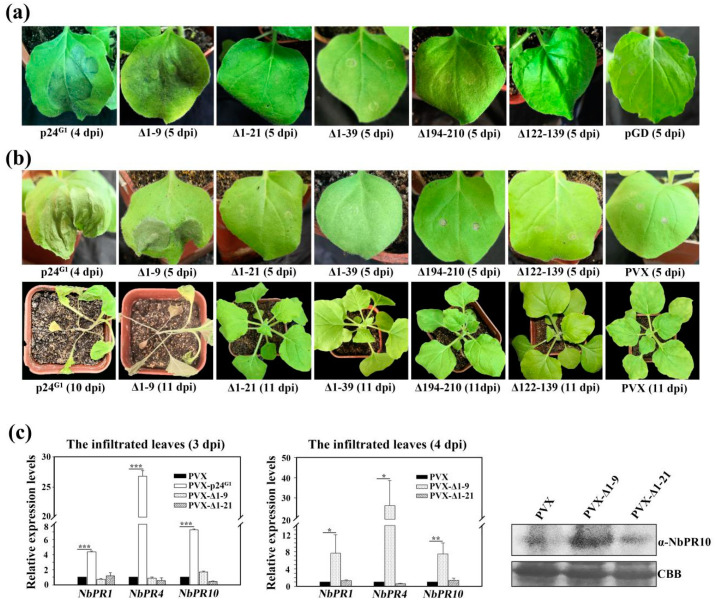
Analysis of the pathogenic activity of p24 of grapevine leafroll-associated virus 1 (p24^G1^) mutants. (**a,b**) Phenotypes of *N. benthamiana* induced by p24^G1^ mutants expressed from pGD (**a**) or PVX (**b**) vector. (**c**) Effects of p24^G1^ mutants on the expression of *NbPR1*, *NbPR4* and *NbPR10* at 3 (left panel) and 4 (middle and right panel) dpi. Infiltrated leaves at 3 and 4 dpi were used for extraction of total RNA and protein. PVX–p24^G1^-infiltrated leaves showing local necrosis at 4 dpi could not be used for analysis. qRT-PCR results are shown as mean ± standard deviation of three independent experiments. Standard deviation is denoted by error bars. * *p* < 0.05, ** *p* < 0.01, *** *p* < 0.001. Right panel indicates NbPR10 accumulation at 4 dpi detected by Western blot using anti-NbPR10 antiserum. CBB staining served as a loading control.

∆1–9, with self-interaction ability and RSS activity, and dimerization-defective mutants with (∆1–21) or without (∆1–39, ∆122–139 and ∆194–210) RSS activity, were selected to assess the effects of RSS activity and dimerization on p24^G1^ function in pathogenesis. Local necrosis was observed in *N. benthamiana* tissues expressing ∆1–9 from the pGD vector at 5 dpi, a one-day delay compared to p24^G1^-expressing leaves. However, the other mutants all lost their ability to elicit a necrotic response in *N. benthamiana* leaves (Figure 7a). These mutants were then expressed from the PVX vector to investigate their effects on the pathogenicity of recombinant PVX viruses. PVX–∆1–9 was able to elicit systemic necrosis, eventually resulting in plant death, but also with a one-day delay: necrosis in the infiltrated patches and apical necrosis were observed at 5 and 7 dpi, respectively, and the whole plant died at 11 dpi, compared to 4, 6 and 10 dpi, respectively, in PVX–p24^G1^-infected plants. In contrast, *N. benthamiana* plants infected with PVX–∆122–139, PVX–∆1–21, PVX–∆1–39 or PVX–∆192–210 all displayed a phenotype similar to that caused by PVX infection (Figure 7b). These results indicated that both RSS activity and self-interaction are required for p24^G1^ function in pathogenesis.

In addition, mutants ∆1–9 and ∆1–21 were further selected to assess their effects on the transcript levels of *PR* genes. As shown in Figure 7c, similar to p24^G1^, ∆1–9 also greatly upregulated the expression of *NbPR1*, *NbPR4* and *NbPR10* in the infiltrated leaves at 4 dpi (middle panel), a one-day delay compared to the PVX–p24^G1^-infiltrated counterparts (left panel). In contrast, no significant difference in the expression of *NbPR1*, *NbPR4* and *NbPR10* was observed between PVX–∆1–21- and PVX-infiltrated leaves at 3 and 4 dpi. Thus, our results revealed a correlation between the pathogenic activity of p24^G1^ and the upregulation of *PR* genes, supporting the notion that bioactive p24^G1^ induces a HR-like response.

## 4. Discussion

Viral RSSs are key components of the counter-defense system, enabling viruses to overcome plant defenses. Therefore, identification of a viral RSS and elucidation of possible mechanisms involved in RNA-silencing suppression contribute to an understanding of the molecular basis underlying viral infection. Here, using an *Agrobacterium* coinfiltration assay, we showed that p24^G1^ encoded by GLRaV-1 is an RSS, blocking local and systemic RNA silencing, and that its suppression activity is comparable to that of TBSV p19 (Figure 1). 

The finding that p24^G1^ self-interacts in vitro and in vivo (Figure 3) supports the notion that oligomerization is a common phenomenon for unrelated RSSs encoded by a variety of plant viruses [9,11,12,13]. Moreover, the fact that the aa 10–210 region, containing all of the α-helices and β-strands, was required for p24^G1^ self-interaction (Figure 3c) also highlights the important role of α-helices and β-strands in dimer formation, as has been demonstrated or suggested for other viral RSSs [9,11,12]. However, in contrast to previous reports for viral RSSs such as CMV 2b and γb of barley stripe mosaic virus, which need to be homodimers to become functional [11,13], our results showed that the dimerization-defective mutant ∆1–21 retains RSS activity, suggesting that the p24^G1^ monomer can suppress RNA silencing.

Viral RSSs have been reported to inhibit RNA silencing through ds siRNA binding [4,7,9,41]. We found that p24^G1^ uses the same strategy; it showed recognition *of* 21-nt ds *siRNA* (Figure 5), similar to that reported for *Tombusvirus* p19, HC-Pro of tobacco etch virus and BYV p21 [7]. Moreover, in contrast to crystal structure predictions that p19 [9] and 2b [12] proteins bind ds siRNA as dimers, the dimerization-defective mutants ∆1–21, ∆1–39 and ∆194–210 all bound 21-nt sRNA duplexes (Figure 5), suggesting that monomeric p24^G1^ is able to bind 21-nt ds siRNA. However, ds siRNA binding is not sufficient for the suppressive function of p24^G1^, because both ∆1–39 and ∆194–210 lost this function (Figure 6). This phenomenon is similar to that reported for RNase3 of sweet potato chlorotic stunt virus [42]. Comparing the numbers of α-helixes and β-strands contained in ∆1–21, ∆1–39 and ∆194–210, it seems that secondary *structural* elements may also be involved in the RSS activity of p24^G1^. Interestingly, mutants ∆1–9, ∆1–21, ∆1–39 and ∆194–210 bound 21-nt sRNA duplexes much more effectively than wt p24^G1^ (Figure 5), although their soluble expression was greatly affected by the mutations (Figure 3d, Figure S5). These results suggest that the deletion of aa 9–39 from the N terminus or aa 16 from the C terminus of p24^G1^ leads to a more favorable conformation for its binding to ds siRNA.

The subcellular localization assay revealed that p24^G1^ mainly accumulates in the nucleus, in agreement with the distribution pattern of some other viral RSSs [16,17,18,19]. Consistent with previous reports for CMV 2b [17], GVX p15 [18] and CVB p12 [19], our data also indicate that the nuclear distribution pattern of p24^G1^ is not essential for its RSS activity, because ∆1–21 showed impaired nuclear localization (Figure 4) but could block RNA silencing (Figure 6). Moreover, similar to the canonical *importin α/β nuclear import* pathway adopted by CMV 2b [43] and p6 of cauliflower mosaic virus (CaMV) [16], p24^G1^ also interacted with NbIMPα1 (Figure 4b), suggesting that the nuclear import of p24^G1^ is mediated by importin α1, although other importin α1-independent nuclear transport pathways cannot be excluded. However, in contrast to the report by Haas et al. [16], where it was shown that monomeric P6 of CaMV can be imported into the nucleus through the importin α pathway, p24^G1^ homodimerization was required for its interaction with nuclear import receptor importin α1 and subsequent nuclear transport (Figure 3 and Figure 4).

Expression of p24^G1^ from the pGD- or PVX-based vectors triggered local necrosis and lethal systemic necrosis in the model plant *N. benthamiana*, respectively, and the enhanced symptoms caused by PVX–p24^G1^ were not correlated with an increase in the titer of PVX (Figure 2). Moreover, the systemic necrosis elicited by PVX–p24^G1^ shared HR features (Figure 2), and there was a correlation between the pathogenic activity of p24^G1^ and the upregulation of *PR* genes (Figure 7c). Therefore, our results demonstrate that biologically active p24^G1^ is a factor in pathogenesis and can elicit a HR-like response in *N. benthamiana*. The HR response is commonly associated with specific recognition of a pathogen avirulence (*avr*) factor by a host *R* gene product [44], and some of viral RSSs have been reported to be elicitors of *R* gene-mediated HR [45,46]. Our finding warrants further investigation into whether p24^G1^ is an *avr* gene encoded by GLRaV-1, which is recognized by an unknown host *R* gene, leading to HR. Our results also revealed that RSS activity is required but insufficient for p24^G1^ to be a factor in pathogenesis, while self-interaction must be preserved, because *dimerization-defective* mutants ∆1–21, ∆1–39, ∆122–139 and ∆194–210 all failed to elicit HR-like necrosis in *N. benthamiana* (Figure 7), even though ∆1–21 retained RSS activity (Figure 6). The importance of self-interaction for p24^G1^ pathogenic activity is consistent with previous reports for CMV 2b [13] and βC1 of tomato yellow leaf curl China betasatellite [47].

Basic aa residues are critical for protein nuclear transport [40], as well as for viral RSS binding of siRNA and suppression of RNA silencing [10,12,27]. p24^G1^ contains a predicted NLS in the aa 122–139 region (Figure S4a) that is rich in basic residues (Figure S3a), and our results showed that mutant ∆122–139 almost lost its nuclear localization, and failed to bind ds siRNA, block RNA silencing and induce a necrotic response in *N. benthamiana* (Figure 4, Figure 5, Figure 6 and Figure 7). These results indicate that the region may act as an NLS, and is also crucial for p24^G1^ binding to siRNA, suppressing RNA silencing and acting as a factor in pathogenesis. Analysis of single and multiple mutations is needed to further evaluate the role of the basic aa clusters in this region.

Viral suppressor proteins are diverse in sequence, structure and function. Our previous works showed that p24 encoded by GLRaV-2 belonging to the same family as GLRaV-1 functions as an RSS [27]. GLRaV-1 p24^G1^ and GLRaV-2 p24 share only 7.62% sequence identity at the aa level, although they have similar molecular weight. Therefore, it is not surprising that the two suppressor proteins display distinct biological features: p24^G1^ localizes in the nucleus (Figure 4) and can suppress RNA silencing as a monomer (Figure 6), whereas p24 accumulates in the cytoplasm and self-interaction is important for p24 functionality [27]. 

In conclusion, our results demonstrate that p24^G1^ of GLRaV-1 localizes in the nucleus and acts as a strong RSS and a factor in pathogenesis. p24^G1^ is able to bind 21-nt ds siRNA, and siRNA binding is required but not sufficient for its suppressive function. p24^G1^ interacts with itself, and dimerization is required for its pathogenic activity and importin α1-mediated nuclear targeting, but not for siRNA binding or RSS activity. The results presented here provide important insights into the molecular mechanisms of GLRaV-1 interactions with plant cells.

## Supplementary Materials

The following are available online at https://www.mdpi.com/1999-4915/12/10/1111/s1, Figure S1: Local necrosis was observed in leaf patches of *N. benthamiana* line 16c coinfiltrated with pGD–p24^G1^/pGD–GFP at 4 dpi, but not in leaves coinfiltrated with pGD–GFP/pGD. Arrows indicate infiltrated leaves; Figure S2: Western blot and RT-PCR results confirm accurate maintenance of p24^G1^ in the viral progeny. The upper noninfiltrated leaves of PVX–p24^G1^- or PVX-infected plants at 4 dpi were used for RNA and protein extraction. Anti-p24^G1^ antiserum was used to detect p24^G1^ accumulation in Western blot analysis. CBB staining served as a loading control. M, DNA marker; Figure S3: Predicted secondary structure of p24^G1^ (a) and schematic representation of deletion mutants of p24^G1^ (b); Figure S4. p24^G1^ mutant ∆122–139 loses ability for homologous interaction. (a) *Bioinformatics analysis* prediction of an NLS in the aa 122–139 region of p24^G1^ and schematic representation of deletion mutant ∆122–139. (b,c) ∆122–139 failed to self-interact and interact with wt p24^G1^ in both yeast (b) and plant (c) cells. For YTHS, ∆122–139 was fused to GAL4 AD and BD. For the BiFC assay, ∆122–139 was fused to YFP^N^ and YFP^C^. Bars = 50 μm. Expression of YFP^N^-tagged p24^G1^ and ∆122–139 detected by Western blot using anti-p24^G1^ antiserum is indicated at the bottom of the fluorescence images. CBB staining served as a loading control. Pairs of BD–p24^G1^/AD–p24^G1^ (b) and p24^G1^–YFP^N^/p24^G1^–YFP^C^ (c) served as positive controls; Figure S5: Yield of purified His-tagged p24^G1^ and its mutants obtained from the *E*. *coli* expression system. M, protein marker. Arrows indicate positions of His-tagged fusion proteins; Table S1: Primers used in this study.
