# Supplementary material for: p24G1 Encoded by Grapevine Leafroll-Associated Virus 1 Suppresses RNA Silencing and Elicits Hypersensitive Response-Like Necrosis in Nicotiana Species
Source: Viruses. 2020 Sep 30;12(10):1111. doi: 10.3390/v12101111 (PMC7601950; doi:10.3390/v12101111)
Supplement: Supplementary file 1 [file viruses-12-01111-s001.zip › Supplementary material/Table S1.docx]

| **Primer** | **Sequence (5’→ 3’)** |
| --- | --- |
| F1 | ATGGCGTCACTTATACCTAG |
| R1 | TCACACCAAATTGCTAGCTA |
| F2 | GCAATACAAGAGGTCCAAACCA |
| R2 | ACTTAGACCGTATGTGCTCGC |
| F3 | GAC*GAATTC*ATGGCGTCACTTATACCTAG |
| R3 | ATC*GGATCC*CTCACACCAAATTGCTAGCTA |
| F4 | GAC*GAATTC*ATGGGAAGTTTCTCCGAACTTTG |
| F5 | GAC*GAATTC*ATGGTATCGAACGCGGATA |
| F6 | CAG*GAATTC*ATGCGATGGTTGGAATTATCGTAC |
| R4 | ATC*GGATCC*CTCAGAGTAAATAATAAGACGA |
| F7 | CA*GGATCC*ATGGCGTCACTTATACCTAG |
| R5 | CT*CTCGAG*CACCAAATTGCTAGCTATAG |
| F8 | AT*GGATCC*ATGGGAAGTTTCTCCG |
| F9 | AA*GGATCC*ATGGTATCGAACGCGGA |
| F10 | CA*GGATCC*ATGCGATGGTTGGAATT |
| R6 | CT*CTCGAG*GAGTAAATAATAAGACGA |
| F11 | AG*CTCGAGCT*ATGGCGTCACTTATACCTAG |
| R7 | TAC*GGATCC*TCACACCAAATTGCTAGCTA |
| F12 | GAC*CTCGAG*CTATGGGAAGTTTCTCCGA |
| F13 | GGA*CTCGAG*CTATGGTATCGAACGC |
| F14 | GGG*CTCGAG*CTATGCGATGGTTGGAATT |
| R8 | GC*GGATCC*TCAGAGTAAATAATAAGACGA |
| F15 | A*GGATCC*ATGGCGTCACTTATACCTAG |
| R9 | AC*GTCGAC*CACCAAATTGCTAGCTATAG |
| F16 | CGT*GGATCC*ATGGGAAGTTTCTC |
| F17 | AAT*GGATCC*ATGGTATCGAACGCGGATA |
| F18 | AAT*GGATCC*ATGCGATGGTTGGAATT |
| R10 | AC*GTCGAC*GAGTAAATAATAAGACGA |
| F19 | CG*CTCGAG*CTATGGCGTCACTTATACCTAG |
| R11 | CG*GGATCC*TCACACCAAATTGCTAGCTA |
| F20 | GCG*ATCGAT*ATGGCGTCACTTATACCTAG |
| R12 | ATT*CCCGGG*TCACACCAAATTGCTAGCTA |
| F21 | GGA*ATCGAT*ATGGGAAGTTTCTCCGAACTTTG |
| F22 | GAG*ATCGAT*ATGGTATCGAACGCGGATAGTGTTC |
| F23 | CGA*ATCGAT*ATGCGATGGTTGGAATTATCGT |
| R13 | ATT*CCCGGG*TCAGAGTAAATAATAAGAC |
| F24 | AT*CTCGAG*CTATGGTTGCACCAACTAGAG |
| R14 | AT*GGATCC*CTAGGCAGCAGCCTTTTGC |
| F25 | ATA*GGATCC*ATGTCGCTGAGGCCGAATTC |
| R15 | ACA*CTCGAG*TGAACTGAAGTTGAATCCTCCT |
| F26 | AA*GGATCC*ATGGCGTCACTTATACCTAG |
| R16 | CT*CTCGAG*TCACCAAATTGCTAGCTATAG |
| F27 | AA*GGATCC*ATGGGAAGTTTCTCCGA |
| F28 | AA*GGATCC*ATGGTATCGAACGC |
| F29 | AA*GGATCC*ATGCGATGGTTGGAATT |
| R17 | CT*CTCGAG*TGAGTAAATAATAAGACGA |
| F30 | AT*GGATCC*ATGGTGAGCAAGG |
| R18 | ATA*CTCGAG*TAAGATCTACCATGTACAGCT |
| F31 | CACAACACAGCCCATAGGGTC |
| R19 | TTAACATCCAGTTCCATACCACTG |
| F32 | CATAACACAGCTCGTGCAGATGTA |
| R20 | ACCTGGAGGATCATAGTTGCAAGAG |
| F33 | GAACAGTCCAGGCTCGCATT |
| R21 | CCGTTACAGTCTCCAGTCTCA |
| F34 | GAAGGTTCTCCTATTAAGTACTTGAAGC |
| R22 | AGCTTGATCTTTGCCTTCACTGTG |
| F35 | AGCTCAAGGGAATTCTCGATG |
| R23 | AACCTTAACCATGTCATCTCCC |
| GFP-sRNA-probe-1 | TGGTGCGCTCCTGGACGTAGCCTTCGGGCATGGCGGACTTGA |
| GFP-sRNA- probe-2 | TCGTTGGGGTCTTTGCTCAGGGCGGACTGGGTGCTCAGGTAG |
| siRNA-1 | GUCACUACUAUGGGUUAUGAG |
| siRNA-2 | CAUAACCCAUAGUAGUGACUG |
| siRNA-3 | AGACCGUGAGGCCAAACUUGGCAU |
| siRNA-4 | GCCAAGUUUGGCCUCACGGUCUAU |
| U6-probe | CGATTTGTGCGTGTCATCCTTG |

Italic sequences indicate restriction sites
