# Supplementary figures and images for: p24G1 Encoded by Grapevine Leafroll-Associated Virus 1 Suppresses RNA Silencing and Elicits Hypersensitive Response-Like Necrosis in Nicotiana Species
Source: Viruses. 2020 Sep 30;12(10):1111. doi: 10.3390/v12101111 (PMC7601950; doi:10.3390/v12101111)

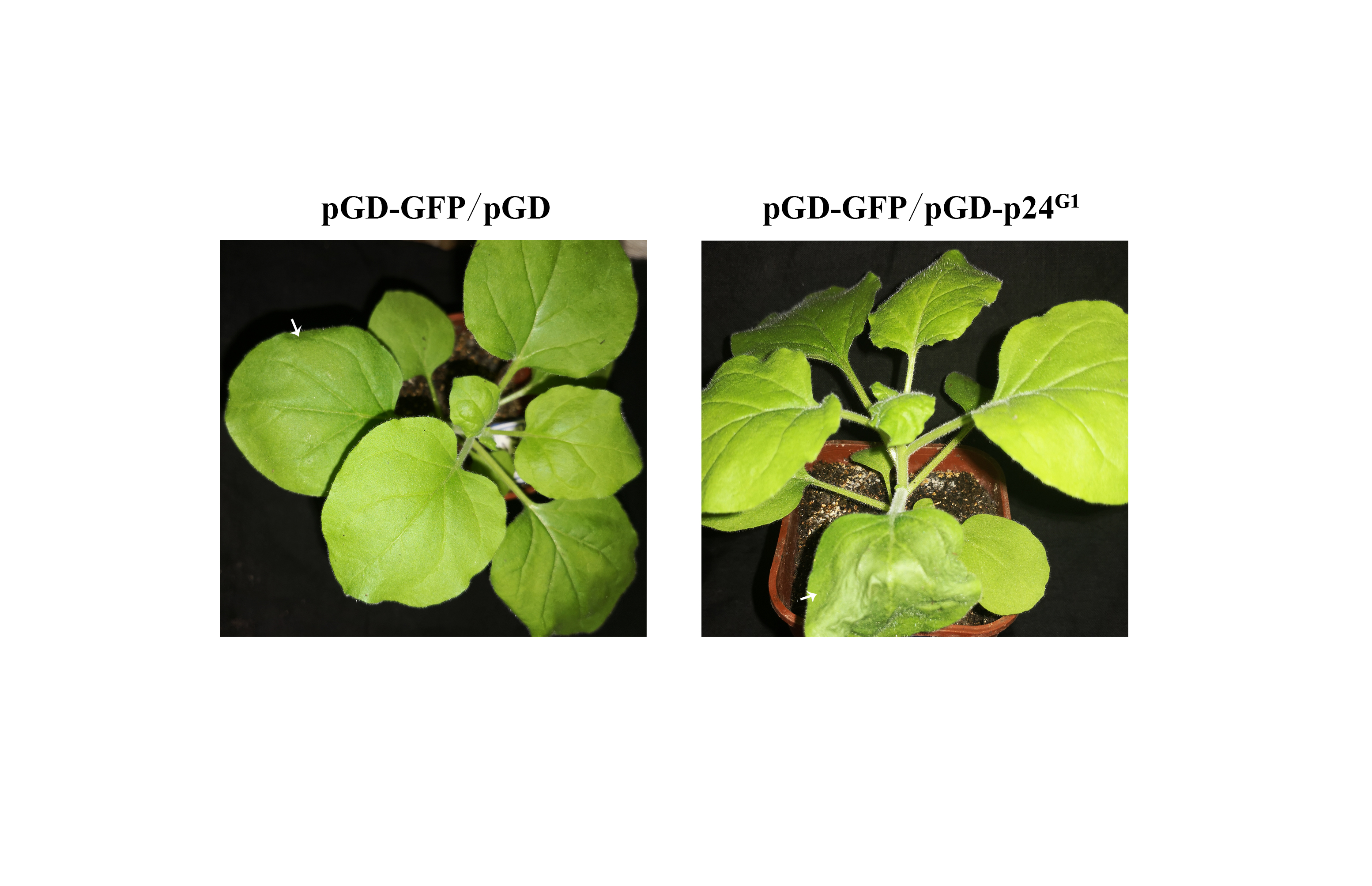

Supplement: Supplementary file 1 [file viruses-12-01111-s001.zip › Supplementary material/Fig S1 .jpg]

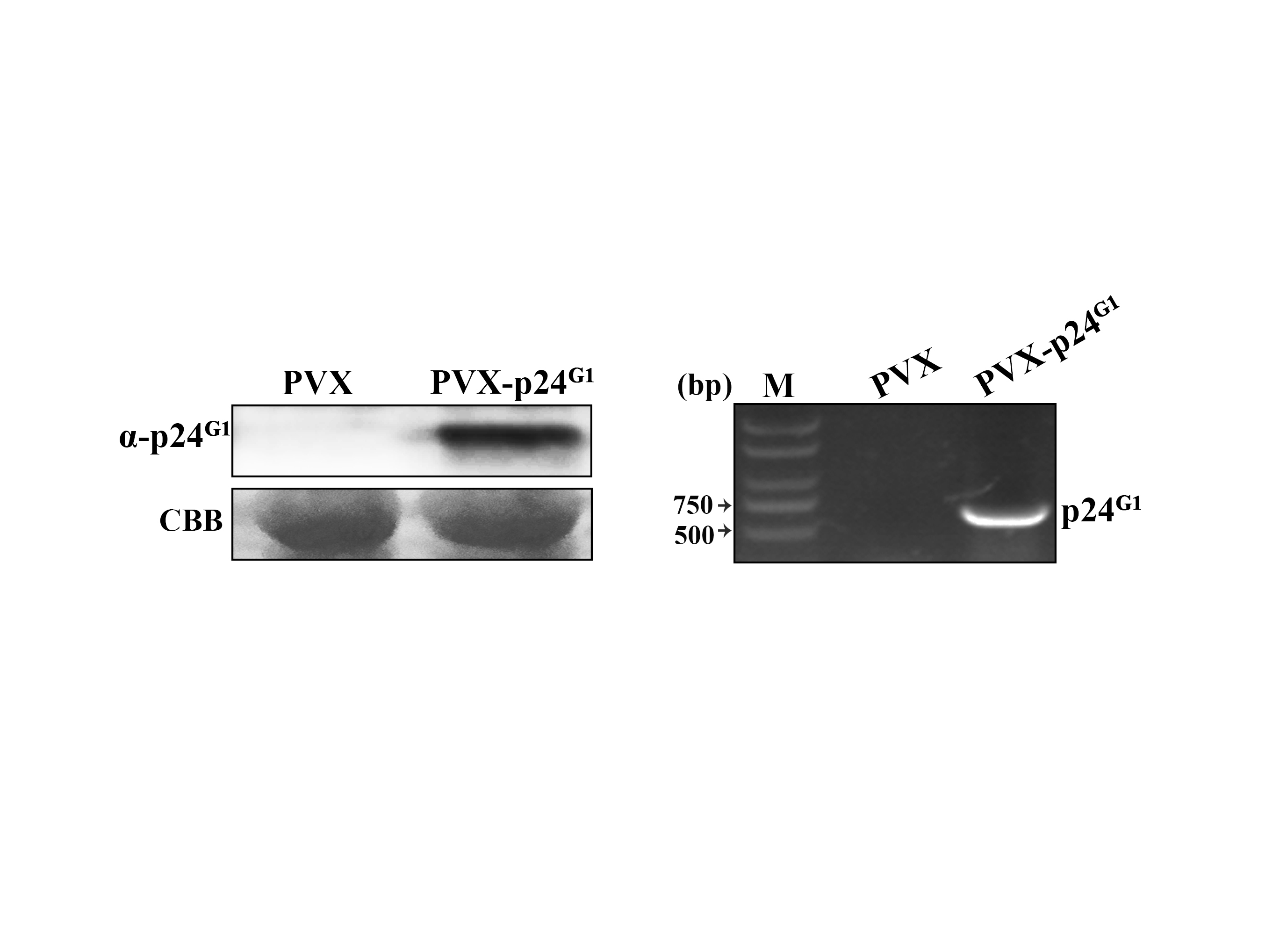

Supplement: Supplementary file 1 [file viruses-12-01111-s001.zip › Supplementary material/Fig S2 .jpg]

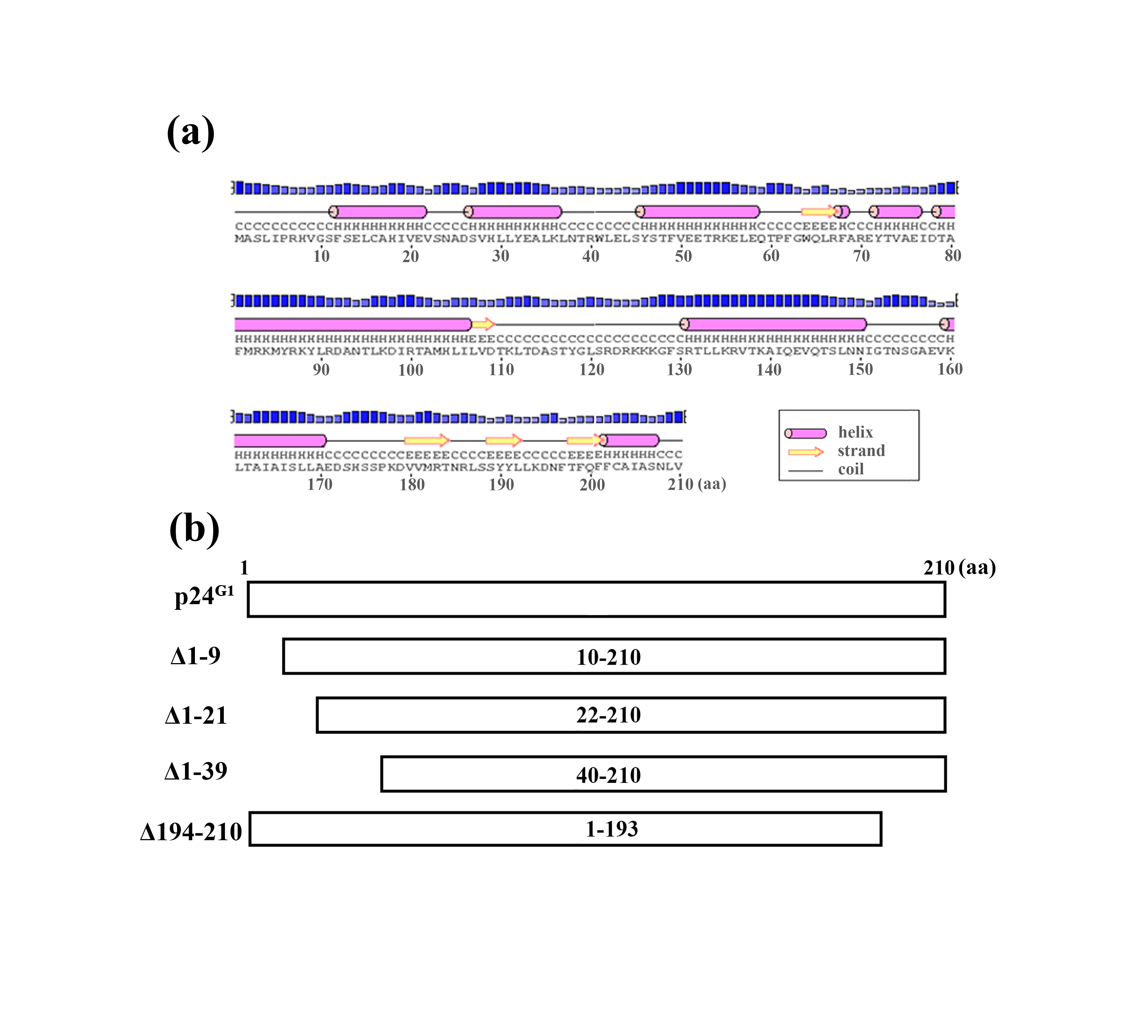

Supplement: Supplementary file 1 [file viruses-12-01111-s001.zip › Supplementary material/Fig S3.jpg]

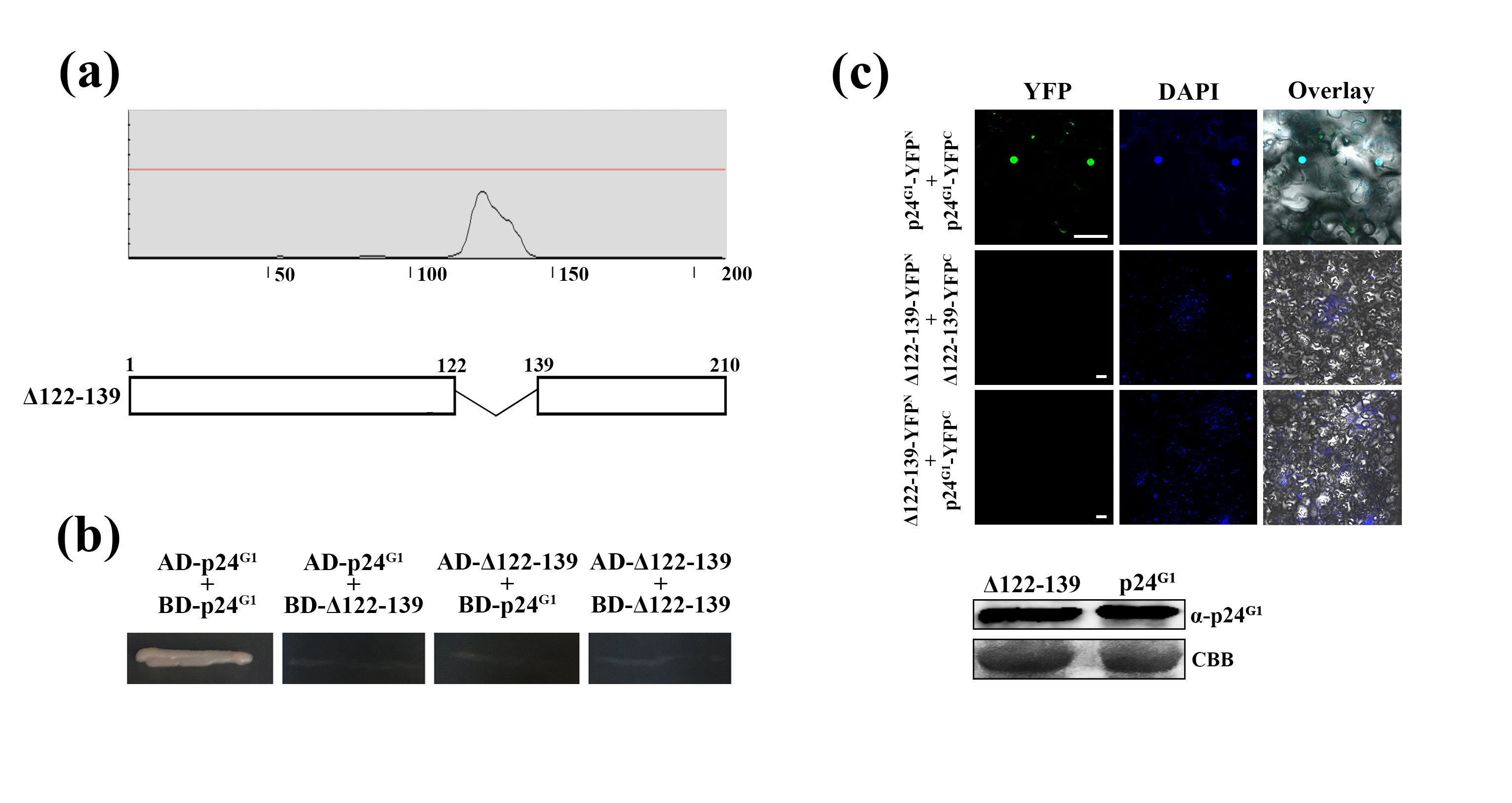

Supplement: Supplementary file 1 [file viruses-12-01111-s001.zip › Supplementary material/Fig S4 .jpg]

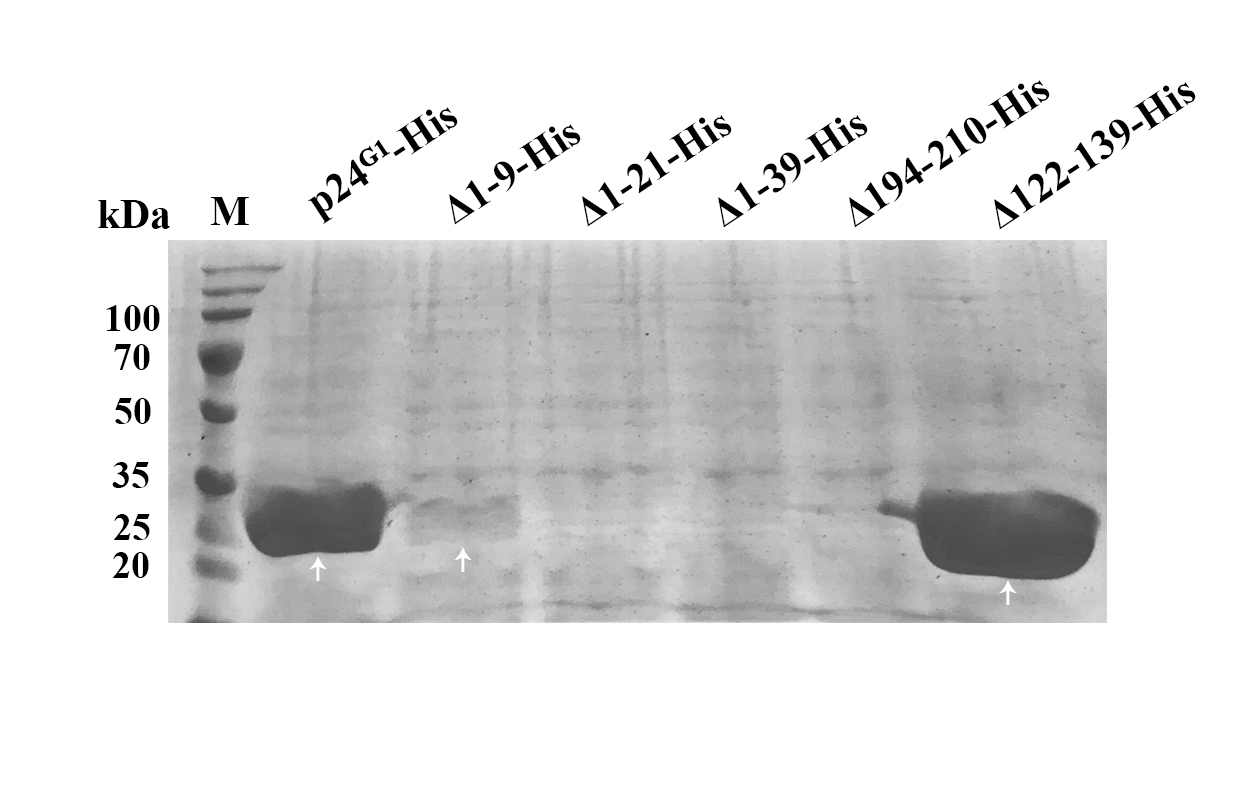

Supplement: Supplementary file 1 [file viruses-12-01111-s001.zip › Supplementary material/Fig S5.jpg]
